# The Radiolabeling of [161Tb]-PSMA-617 by a Novel Radiolabeling Method and Preclinical Evaluation by In Vitro/In Vivo Methods

**DOI:** 10.21203/rs.3.rs-3415703/v1

**Published:** 2023-10-30

**Authors:** EMRE UYGUR, Ceren Sezgin, Yasemin Parlak, Kadriye Busra Karatay, Bilal Arikbasi, Ugur Avcibasi, Turkay Toklu, Sabri Barutca, Coskun Harmansah, Tevfik Sinan Sozen, Stephan Maus, Howard Scher, Omer Aras, Fikriye Gul Gumuser, Fazilet Zumrut Biber Muftuler

**Affiliations:** Manisa Celal Bayar University: Manisa Celal Bayar Universitesi; Manisa State Hospital: Manisa Devlet Hastanesi; Manisa Celal Bayar University: Manisa Celal Bayar Universitesi; Ege University Institute of Nuclear Sciences: Ege Universitesi Nukleer Bilimler Enstitusu; Manisa State Hospital: Manisa Devlet Hastanesi; Manisa Celal Bayar Üniversitesi: Manisa Celal Bayar Universitesi; Yeditepe University: Yeditepe Universitesi; Adnan Menderes Üniversitesi Tıp Fakültesi: Adnan Menderes Universitesi Tip Fakultesi; Ege University: Ege Universitesi; Gazi University Faculty of Medicine: Gazi Universitesi Tip Fakultesi; Saarland University Hospital and Saarland University Faculty of Medicine: Universitatsklinikum des Saarlandes und Medizinische Fakultat der Universitat des Saarlandes; Memorial Sloan-Kettering Cancer Center Inpatient Hospital and Main Campus: Memorial Sloan Kettering Cancer Center; Memorial Sloan-Kettering Cancer Center Inpatient Hospital and Main Campus: Memorial Sloan Kettering Cancer Center; Manisa Celal Bayar University: Manisa Celal Bayar Universitesi; Ege University Institute of Nuclear Sciences: Ege Universitesi Nukleer Bilimler Enstitusu

**Keywords:** Terbium-161 [161Tb]Tb, PSMA-617, prostate cancer

## Abstract

**Background:**

Prostate cancer (PC) is the most common type of cancer in elderly men, with a positive correlation with age. As resistance to treatment has developed, particularly in the progressive stage of the disease and in the presence of microfocal multiple bone metastases, new generation radionuclide therapies have emerged. Recently, [^161^Tb], a radiolanthanide introduced for treating micrometastatic foci, has shown great promise for treating prostate cancer.

**Results:**

In this study, Terbium-161 [^161^Tb]Tb was radiolabeled with prostate-specific membrane antigen (PSMA)-617 ([^161^Tb]-PSMA-617) and the therapeutic efficacy of the radiolabeled compound investigated *in vitro* and *in vivo*. [^161^Tb]-PSMA-617 was found to have a radiochemical yield of 97.99 ± 2.01% and was hydrophilic. [^161^Tb]-PSMA-617 was also shown to have good stability, with a radiochemical yield of over 95% up to 72 hours. *In vitro*, [^161^Tb]-PSMA-617 showed a cytotoxic effect on LNCaP cells but not on PC-3 cells. *In vivo*, scintigraphy imaging visualized the accumulation of [^161^Tb]-PSMA-617 in the prostate, kidneys, and bladder.

**Conclusions:**

The results suggest that [^161^Tb]-PSMA-617 can be an effective radiolabeled agent for the treatment of PSMA positive foci in prostate cancer.

## Background

Prostate cancer is the second most prevalent cancer among men and the fifth leading cause of cancer-related deaths in males globally (Arnold et al., 2015; He et al., 2021). The management of prostate cancer at disease presentation is based on disease extent, defined by states(Scher & Heller, 2000) ranging from clinically localized disease to clinical metastases in need of or having been treated with androgen deprivation therapy. Androgen deprivation therapy remains the first-line standard systemic approach for tumors at a high risk of metastasizing or that have already spread to distant sites and can be given in the form of monotherapy or in combination with recently approved next-generation inhibitors of androgen signaling to produce a dramatic response. However, androgen deprivation therapy is not curative and virtually all cancers treated with this therapy progress to a metastatic castration resistant state which is lethal for most patients. Hence, in the ever-evolving landscape of prostate cancer treatment, significant strides have been made to further improve patient outcomes, including the development of approved agents like taxanes and radium which have been pivotal in managing this complex disease since their introduction (Corn et al., 2019). The field has now further transitioned into the era of precision medicine, marked by the approval of poly ADP ribose polymerase (PARP) inhibitors and the recognition of microsatellite instability alterations as promising therapeutic targets (Fujimoto et al., 2021); further, prostate specific membrane antigen (PSMA)-directed approaches are emerging as an especially potent treatment strategy (Kratochwil, Giesel, et al., 2016).

Collectively, advancements to date in the management of prostate cancer have laid the foundation for the next generation of theranostic PSMA-directed approaches, with terbium (Tb) poised to play a central role (Al-Ibraheem et al., 2023; Müller, Singh, et al., 2019). PSMA is a glycoprotein found on the surface of cells. While it is naturally expressed in normal prostate tissue, it is significantly upregulated or overexpressed in cases prostate cancer. Studies report that PSMA expression level is associated with disease stage and the risk of progression (Kratochwil, Giesel, et al., 2016).

In terms of PSMA-targeted radionuclide therapy, various clinical studies have reported on the use of [^177^Lu]-PSMA-617, [^225^Ac]-PSMA-617, and [^161^Tb]-PSMA-617 to treat metastatic castration resistant prostate cancer (Baum et al., 2016; Fendler et al., 2017, 2019; Feuerecker et al., 2021; Gourni et al., 2017; Kessel et al., 2019; Kratochwil, Bruchertseifer, et al., 2016; Kratochwil et al., 2017, 2018; Kratochwil, Giesel, et al., 2016; Rahbar et al., 2017; Sathekge et al., 2019, 2020; Violet et al., 2020; Yadav et al., 2020). The use of [^177^Lu]Lu as a theranostic agent has shown promising results (Baum et al., 2016; Fendler et al., 2017; Gourni et al., 2017). It is effective in prolonging the lives of patients, particularly in cases with larger lesions. However, it’s important to note that the energy released by [^177^Lu]Lu may not completely eliminate microscopic disease, highlighting the need for complementary treatments or therapies to address residual or smaller lesions (Kessel et al., 2019; Rahbar et al., 2017). As such, the use of [^225^Ac]Ac has also been investigated (Feuerecker et al., 2021; Kratochwil, Bruchertseifer, et al., 2016; Kratochwil, Giesel, et al., 2016; Müller, Umbricht, et al., 2019). Of note, the first studies on the use of PSMA in radioligand-based therapy focused on its use for nuclear imaging and radioactive iodine therapy. PSMA ligands with various chelators were only later developed to enable their use with different radiometals for imaging and therapeutic purposes (Fendler et al., 2017). Currently, PSMA I&T (Imaging and Therapy) and PSMA-617 equipped with a DOTAGA and DOTA chelator, respectively, are used in the clinic for targeted radioligand therapy of metastatic castration resistant prostate cancer (Kratochwil, Giesel, et al., 2016; Sathekge et al., 2020; Violet et al., 2020; Yadav et al., 2020). For end-stage patients without other treatment options, PSMA ligands radiolabeled with [^177^Lu]Lu (T_1/2_ = 6.65 d; Eβ _av_ = 134 keV; E_γ_ = 113 keV, I = 6.117%, E_γ_ = 208 keV, I = 10.36%) are used [5], and [^225^Ac]-PSMA-617 has been used in some special cases (Feuerecker et al., 2021; Kratochwil, Bruchertseifer, et al., 2016; Sathekge et al., 2019; Yadav et al., 2020).

More recently, the radiolanthanide [^161^Tb]Tb has been introduced for therapeutic applications because it emits β^−^particles (E_β av_ = 154 keV) as well as γ-radiation (E_γ_ = 49 keV, I = 17.0%; E_γ_ = 75 keV, I = 10.0%) that are suitable for therapeutic purposes and single-photon emission computed tomography (SPECT), respectively (Müller, Umbricht, et al., 2019). [^161^Tb]Tb decays to the stable ^161^Dy with a half-life of 6.89 days (Collins et al., 2022). Also, [^161^Tb]Tb is very similar to [^177^Lu]Lu in terms of radiochemical properties, although the γ-radiation emitted by [^161^Tb]Tb is of a lower energy. In addition, the most important advantage of [^161^Tb]Tb is that it emits a significant number of low energy conversions and auger electrons comparison with [^177^Lu]Lu. This holds great promise for the treatment of prostate cancer that has progressed to disease with multiple metastases of various sizes (Borgna, Barritt, et al., 2021; Grünberg et al., 2014). In Hindié et al.’s study (Hindie et al., 2016), Monte Carlo simulations comparing [^177^Lu]Lu with [^161^Tb]Tb showed that the effect of [^161^Tb]Tb was 3.6 and 1.8 times that of [^177^Lu]Lu in a 10-μm cell and 1.8 times 100-μm micrometastasis, respectively. Some studies already indicate that [^161^Tb]Tb outperforms other clinically used ([^177^Lu]Lu, [^90^Y]Y) and non-standard therapeutic radionuclides ([^47^Sc]Sc, [^67^Cu]Cu) in terms of dose delivery to small lesions (Fendler et al., 2017; Gourni et al., 2017; Hindie et al., 2016).

In this study, the radiopharmaceutical potential of [^161^Tb]-PSMA-617 radiolabeled with new method (Patent Id: TP23–1225) was investigated for the first time in Turkey through *in vitro* and *in vivo* methods.

## Methods

### Chemicals and Materials

PSMA-617 was purchased from EDH Health Co (İstanbul, Turkey). Thin-layer chromatography paper (ITLC-silica), ammonium acetate, n-octanol, methanol and acetonitrile were purchased from Merck Chemical (İstanbul, Turkey). Minimum Essential Medium (MEM) non-essential amino acid, Dulbecco’s Modified Eagle Medium (DMEM), MEM Eagle, Roswell Park Memorial Institute (RPMI) 1640 medium, sodium bicarbonate, sodium pyruvate, fetal bovine serum (FBS), L-glutamine, penicillin/streptomycin, trypan blue, phosphate buffer solution, and trypsin ethylenediaminetetraacetic acid (EDTA) were purchased from Biological Industries (Ankara, Turkey). [^161^Tb]TbCl_3_ was supplied by Terthera (Breda, Netherlands). PC-3 and LNCaP cells were obtained from the American Type Culture Collection (ATCC, Rockville, MD, USA)

### Radiolabeling and Quality Control

A new radiolabeling method was developed by optimizing the radiolabeling of PSMA-617 with [^161^Tb]Tb according to the literature (Al-Ibraheem et al., 2023; Müller, Umbricht, et al., 2019). Specifically, 1 mL sodium acetate buffer (labelling buffer) and 185 MBq [^161^Tb]TbCl_3_ were added into a tube containing 50 μL ascorbic acid and the reaction mixture (pH 4.5) was incubated at 95°C for 10 min. Then, 25 μL of PSMA-617 was added to the mixture. The mixture was incubated in a hot pot at 95°C for ~ 25 min and subsequently cooled at room temperature.

Quality control studies were carried out using radio-TLC, with silica gel TLC strips and 3 mobile phases (Solvent 1: ammonium acetate (1M): methanol (1:1 v/v); Solvent 2: 100% ACN; and Solvent 3: 65 mL of solution A (2.94 g trisodium-citrate-dihydrate solved in 100 mL water) + 35 mL of solution B (2.10 g citric acid-monohydrate solved in 100 mL water)). Radio-TLC measurements were accomplished using a Perkin Elmer Cyclone Storage System (Massachusetts, USA) and a TLC scanner (Bioscan AR-2000 Scanner, Berlin, Germany). A low-pressure gradient high-performance liquid chromatography (HPLC) system [quaternary pump (LC-10ATvp), an NaI(Tl) radioactivity detector (Gabi Star, Raytest Angleur, Belgium), an autosampler (SIL-20A HT), a diode array detector (DAD; SPD-M20A), a fraction collector (FRC-10A), and a column (RP-C18; 5 lm, 250 4.6 mm I.D., ODS GL Sciences, Tokyo, Japan)] was also used, with methanol/dH_2_O (v/v, 80:20) as the mobile phase and a flow rate 1 mL/min. Radioactivity of the radiolabeled compound ([^161^Tb]-PSMA-617) was confirmed using an NaI (Tl) detector (Gabi Star, Raytest, Belgium) at 210–254 nm wavelengths in the HPLC system.

### Stability Studies

[^161^Tb]-PSMA-617, i.e., PSMA-617 radiolabeled with [^161^Tb]Tb under optimum conditions as confirmed by quality control studies, was dropped (2.5 μL) onto TLC plates at 1, 2, 4, 24, 48 and 72 hours, respectively. TLC silica gel strips were run in the optimum bath, i.e., Solvent 1, and additional quality control studies were carried out using radio-TLC. In addition, the variation of the % radiochemical yield versus time was analyzed.

### Lipophilicity Studies

300 μL of n-octanol and 300 μL of ultrapure water were placed in a centrifuge tube, 150 μL of [^161^Tb]-PSMA-617 was added, and the whole mixture was vortexed for 1 min. Then, the upper and lower phases were separated by centrifugation at 1000 rpm for 30 min. 150 μL of these phases were sampled and a Cd(Te) (RAD-501, Isın Electronics, Izmir, Turkey) detector was used to measure the radioactivity between phases. LogP, i.e., lipophilicity, values were then calculated using the formula log (CPS n-octanol phase/CPS phosphate buffer phase).

#### In Vitro Cell Culture Studies

PC3 cells were grown in DMEM, 2 mM of glutamine, 1.5 g/L sodium bicarbonate, 0.1 mM of non-essential amino acids, 1 mM of sodium pyruvate, and 10% of FBS. Meanwhile, LNCaP cells were grown in RPMI 1640 medium, 2 mM of glutamine, 1.5 g/L of sodium bicarbonate, 0.1 mM of non-essential amino acids, 1 mM of sodium pyruvate, and 20% of FBS. Cryotubes in a nitrogen tank were opened and cells were grown in appropriate media and passaged to reach the number of cells required. Sufficiently proliferated cells were removed using trypsin-EDTA solution and seeded in 24- or 96-well plates and kept at 37 ~C and 5% CO_2_ until use in further studies.

### MTT Tests

Solutions containing [^161^Tb]-PSMA at different concentrations corresponding to 1, 2, 4, and 8 μg of PSMA per well and 0.2, 0.4, 0.8, and 1.6 mCi activity were added to PC3 and LNCaP cells seeded in 96-well plate (104 cells per well). As a negative control, cell-free medium was added to the wells. Subsequently, the 96-well plate was incubated at 37 °C in 5% CO_2_ environment for 24 hours. At the 24th hour, 10 μL of MTT solution was added to each well and the 96-well plate was kept under the same conditions for another 4 hours. At the end of those 4 hours, the 96-well plate was read by a spectrophotometer at 570 nm wavelength and the absorbance value for each well was determined. Viability (%) values were calculated using the following formula: viability = (measured absorbance value/control value) × 100. The absorbance of the negative control was accepted as zero.

### Incorporation

In order to determine the uptake efficiency of [^161^Tb]-PSMA-617 on cell lines, cells belonging to both cell lines in the experimental and study groups were seeded in 24-well culture dishes with 5 × 10^3^ cells and 0.5 mL of medium in each well. The time parameters to be examined in the study were determined as 1, 2, 4, 8, and 24 h. Media containing [^161^Tb]-PSMA-617 (4.625 MBq / 0.625 μg PSMA) were added to each well. In the experimental study, each plate’s culture medium containing 4.625 MBq [^161^Tb]TbCl_3_ was added as a control group. At 1, 2, 4, 8, and 24 hours, the initial amount of radioactivity (A_0_) per well was determined by counting the activity of the labeled medium on the cells in each well using a Cd(Te) detector. When the planned incubation periods were completed, the labeled media in the wells were removed and the cells were washed with sterile PBS. 500 μL of PBS was added to each well and radioactivity counting (A_1_) was performed again. The A1 and A0 values detected for the radiolabeled compound and free [^161^Tb] were ratioed to determine the % binding efficiency (A_1_/A_0_ * 100). In each cell line, all time parameters were performed in 3 replicates to reach enough repetitions of the study.

#### In Vivo Studies

Male Wistar Albino rats were used for scintigraphy imaging (n = 3) and for biodistribution studies (n = 12) of [^161^Tb]PSMA-617 within the scope of *in vivo* studies. Ethics committee approval for *in vivo* studies were obtained from the Manisa Celal Bayar University Local Animal Experiments Ethics Committee (approval date, February 28, 2023; protocol number 77.637.435–254). The male Wistar Albino rats were obtained from Manisa Celal Bayar University Experimental Animal Center.

### Scintigraphy Imaging

Scintigraphy imaging studies were performed on male Wistar Albino rats (n = 3). Rats were administered 2 mL/kg of the anesthetic agent [2.5 mL of ketamine (80 mg/kg) + 0.5 mL of SF + 2 mL xylazine (4 mg/kg)]. Anaesthetized rats were intravenously injected with [^161^Tb]-PSMA-617 (~ 33.3 MBq) via the tail vein. Scintigraphy images were obtained with a dual-head gamma camera (Infinia, GE, Tirat Hacermel, Israel), with a low-energy, high-resolution collimator, imaging the whole body. After the injection of [^161^Tb]-PSMA-617, static images were obtained at different time intervals (0.5, 1, 2, 4, 24 hours after injection) with a 256 × 256 matrix. CT images were also obtained.

### Biodistribution Studies

Biodistribution studies were performed in 12 rats at the 1st, 4th, 24th, and 48th hour (n = 3 rats for each time point) after the injection of [^161^Tb]-PSMA-617 into the tail vein. The activity of the injectors in the full state just before injection and the activity of the injectors in the empty state after injection was measured using a dose calibrator (CRC-55t, Capintec, New Jersey, USA) and the net mean injection activity was determined to be 37 MBq (1 mCi). After injection, the rats were sacrificed under anesthesia and the blood, the heart, the lung, the liver, the kidney, the small intestine, the large intestine, the stomach, the spleen, the pancreas, the muscle, the testis, the prostate, the fat, the bladder, the brain, the salivary glands, the thyroid, the skin, and the stool parts were removed. Extracted samples were placed in pre-tightened containers and weighed with a precision balance, and then activity counts were obtained using a Cd(Te) detector. Activity values for each organ/tissue were calculated in Microsoft Excel, accounting for time corrections, and the % ID/g-time graph of each organ/tissue was drawn.

### Statistical Analysis

Mean radiochemical yields and standard deviations were calculated, with three replicates conducted for each parameter. For *in vitro* cell culture studies, the Graph Pad program was utilized to conduct one-way analysis of variance (ANOVA) and Pearson correlation statistics. Significance testing was conducted at a confidence level of 95% (p < 0.05) to determine if there was a significant difference between the intake and uptake values.

## Results and Discussion

### Results

**Figure 1.**
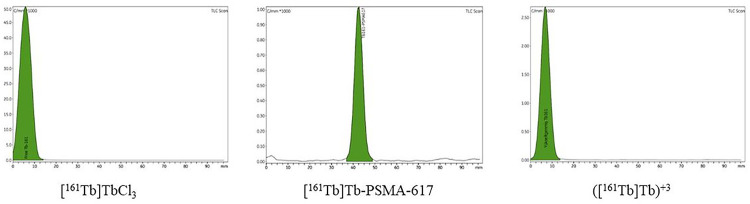
TLRC Chromatograms.

**Figure 2.**
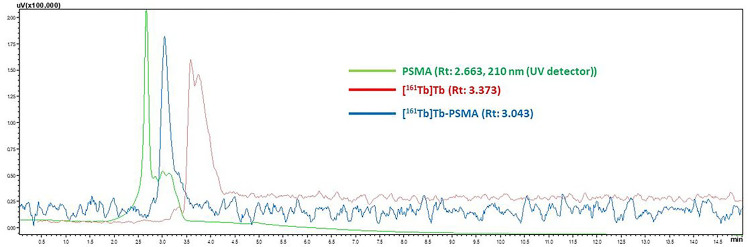
HPLRC Chromatograms.

**Figure 3.**
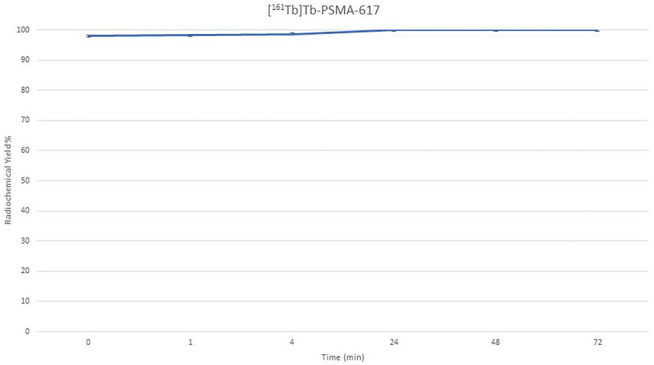
Stability of the [^161^Tb]Tb-PSMA-617

In this study, Terbium-161 [^161^Tb]Tb was radiolabeled with PSMA-617 to yield [^161^Tb]-PSMA-617 and the therapeutic efficacy of the radiolabeled compound investigated *in vitro* and *in vivo*. The radiochemical yield of [^161^Tb]-PSMA-617 was determined using radio-TLC and HPLC. Based on the radio-TLC chromatograms presented in Fig. 1, the R_f_ (Relative Front) values of [^161^Tb]Tb, [^161^Tb]Tb^+ 3^, and [^161^Tb]-PSMA-617 were 0.053, 0.043, and 0.073, respectively. Conversely, according to the HPLRC chromatograms seen in Fig. 2, the retention times of PSMA-617, [^161^Tb]Tb, and [^161^Tb]-PSMA-617 were 2.663, 3.373, and 3.043 minutes, respectively. The radiochemical yield of [^161^Tb]-PSMA-617 was 97.98% ± 2.01 (n = 6) based on these measurements. Figure 3 shows that the [^161^Tb]-PSMA-617 molecule maintained its stability for 72 hours with a yield over 95%. In terms of lipophilicity, the logP value of [^161^Tb]-PSMA-617 was − 2.15 ± 0.31, with the negative logP value indicating that the [^161^Tb]-PSMA-617 molecule is hydrophilic.

**Figure 4.**
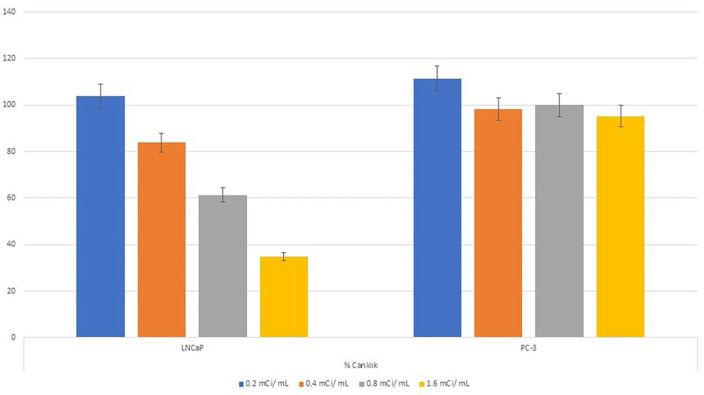
24 h cell viability graph of [^161^Tb]Tb-PSMA-617 on PC-3 and LNCaP cell lines.

**Figure 5.**
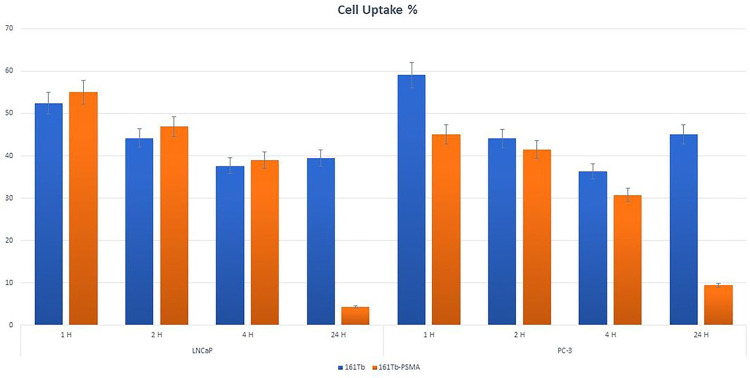
Cell uptake graph of [^161^Tb]TbCl3 and [^161^Tb]Tb-PSMA-617(P≤ 0.05).

The cytotoxicity graph based on LNCaP and PC3 viability values is given in Fig. 4. It was observed that [^161^Tb]-PSMA-617 at increased concentrations showed a cytotoxic effect on LNCaP cells, while no cytotoxic effect was observed on PC-3 cells. The graph of cell incorporation results is given in Fig. 5, showing that the uptake rate of [^161^Tb]-PSMA in LNCaP and PC3 cells was approximately 40% for 4 hours. According to 2-way ANOVA for the optimum time of cell retention, a significant difference was found between [^161^Tb]TbCl_3_ and [^161^Tb]-PSMA-617 in LNCaP and PC3 cells.

**Figure 6.**
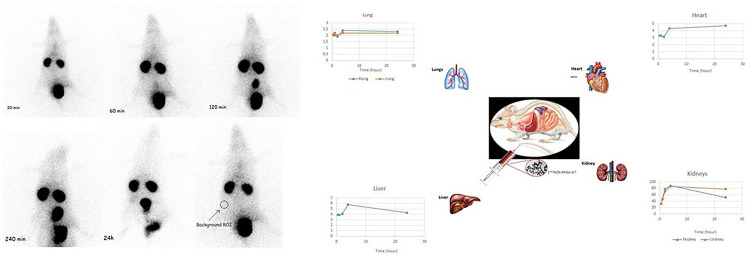
Scintigraphy images in the different time periods of the [^161^Tb]Tb-PSMA-617

Scintigraphy imaging visualized the accumulation of [^161^Tb]-PSMA-617 in the prostate, kidneys, and the bladder. Static images of [^161^Tb]-PSMA-617 in rats demonstrated that substantial tracer accumulation was present in the kidneys at 30 min, as seen in Fig. 6. In addition, [^161^Tb]-PSMA-617 activity in the abdominal and chest region also increased with time. [^161^Tb]-PSMA-617 activity was almost entirely excreted after 4 h by renal excretion.

**Figure 7.**
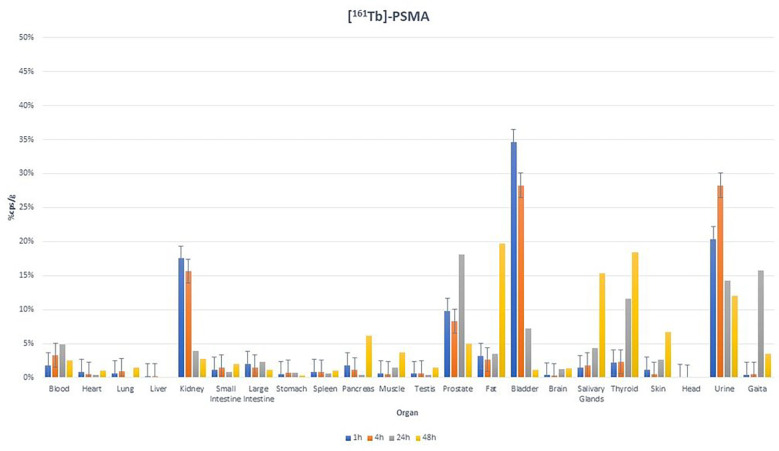
Biodistribution of [^161^Tb]-PSMA-617 On Albino Wistar Rats (n=3)

The findings stemming from our investigation of the biodistribution (Fig. 7) patterns of [^161^Tb]-PSMA-617 in Albino Wistar rats revealed a notable concentration of the compound within a 24-hour timeframe in the renal, vesicular, and urinary compartments. This specific inclination toward renal tissues underscores the dominant route of excretion for [^161^Tb]-PSMA-617 being through the kidneys as noted above. Hematological dynamics displayed an initial surge over a 24-hour period, followed by a subsequent reduction at the 48-hour mark. At the 24-hour time point, a marked increase in fecal content was observed, while at the subsequent 48-hour time point, a statistically significant elevation was noted in specific anatomical sites, including the pancreas, musculature, adipose tissue, salivary glands, and thyroid.

## Discussion

There is increasing interest worldwide in the use of Tb radioisotopes in nuclear medicine applications for cancer therapy and diagnosis (Al-Ibraheem et al., 2023; Borgna, Barritt, et al., 2021; Borgna, Haller, et al., 2021; Cassells et al., 2021; De Jong et al., 1995; Favaretto et al., 2021; Grünberg et al., 2014; Hindie et al., 2016; Müller et al., 2014; Müller, Singh, et al., 2019; Müller, Umbricht, et al., 2019). Particularly, promising results have been reported concerning the potential of [^161^Tb]-radiolabeled compounds for radionuclide therapy (Al-Ibraheem et al., 2023; Cassells et al., 2021; Müller, Umbricht, et al., 2019). In this study, Terbium-161 [^161^Tb]Tb was radiolabeled with PSMA-617 to yield [^161^Tb]-PSMA-617 and the therapeutic efficacy of the radiolabeled compound investigated *in vitro* and *in vivo*. The radiochemical yield is an important parameter for radiopharmaceuticals and is expected to be over 95%. In this study, the radiochemical yield of [^161^Tb]-PSMA-617 was 97.98% ± 2.01 (n = 6).

[^161^Tb]-PSMA-617 molecule maintained its stability for 72 hours with a yield over 95%. [^161^Tb]Tb and [^177^Lu]Lu are both radiolanthanides with similar chemical properties, allowing them to form stable radiometal complexes through chelation with DOTA chelator. This means that [^161^Tb]Tb can be used with the same DOTA-functionalized biomolecules currently employed with [^177^Lu]Lu. The convenience of [^161^Tb]Tb being commercially available in dilute hydrochloric acid solution, like [^177^Lu]Lu, enables the utilization of identical labeling protocols for both radionuclides. Preliminary investigations have also shown comparable stability of radioligands, regardless of whether they are labeled with [^161^Tb]Tb or [^177^Lu]Lu (Borgna, Barritt, et al., 2021; Gracheva et al., 2019; Müller et al., 2014). The stability of the radioligand [^161^Tb]-PSMA-617 is not significantly affected by the emitted conversion and Auger electrons, since its radiolytic decay is due to its behavior, similar to that of [^177^Lu]-PSMA-617.

Our result in this study that [^161^Tb]-PSMA-617 has a radiochemical yield of 97.98% ± 2.01 is similar to the radiochemical yield of 98% reported in Müller et al’s study (Müller, Umbricht, et al., 2019). In Müller et al.’s study, PSMA-617 labeled with [^161^Tb]Tb ≥ 98% radiochemical purity and specific activities up to 100 MBq/nmol. While [^161^Tb]-PSMA-617 remained stable (> 98%) for 1 hour during incubation, radiolytic degradation occurred after. To avoid degradation, [^161^Tb]-PSMA-617 was maintained in the presence of L-ascorbic acid, where it showed stability (≥ 98%) for up to 24 hours without degradation. In our study which used a new method to radiolabel PSMA-617 with [^161^Tb]Tb, optimized based on the existing literature, the use of L-ascorbic acid was also essential to ensure the stability of [^161^Tb]-PSMA-617. According to our results, [^161^Tb]-PSMA-617 was stable for 72 hours in the presence of L-Ascorbic acid. Of note, [^161^Tb]-PSMA-617 in Al-Ibraheem et al.’s study also required the use of L-ascorbic acid to ensure stability (Al-Ibraheem et al., 2023).

In this study, the logP value of [^161^Tb]-PSMA-617 was − 2.15 ± 0.31, with the negative logP value indicating that the [^161^Tb]-PSMA-617 molecule is hydrophilic. Meanwhile, a lipophilicity value of − 3.90 ± 0.1 was reported in Müller et al.’s study (Müller, Umbricht, et al., 2019). The difference in lipophilicity values is thought to be due to the equipment used; whereas Cd(Te) detector was used in this study, Müller et al. obtained measurements with a Perkin Elmer, Wallac Wizard 1480 Gamma Counter.

According to the cytotoxicity graph, [^161^Tb]-PSMA-617 at increased concentrations showed a cytotoxic effect on LNCaP cells, while no cytotoxic effect was observed on PC-3 cells. This can be attributed to the fact that LNCaP cells are androgen receptor cells, and PSMA-617 exhibits higher affinity towards these cells. On the other hand, PC-3 cell lines are androgen receptor-negative cells, which may explain their relatively lower survival compared to LNCaP cells. In terms of cytotoxicity, the results obtained in our study were also similar to those in Müller et al.’s study (Müller, Umbricht, et al., 2019) which demonstrated *in vitro* that the viability and survival of PSMA-positive PC-3 PIP tumor cells decreased corresponding to the administered activity concentration of [^161^Tb]-PSMA-617. Further, Müller et al. found that [^161^Tb]-PSMA-617 was significantly more effective than [^177^Lu]-PSMA-617 in decreasing tumor cell viability (at an activity concentration of 0.1–10 MBq/mL) and survival (at an activity concentration of 0.05–5.0 MBq/ML (P < 0.05 for both). Also, the average energy absorbed by tumor cells was also 3.2–4.2 times higher for [^161^Tb]-PSMA-617 than [^177^Lu]-PSMA-617 in their MTT experiments.

According to the graph of cell incorporation, the uptake rate of [^161^Tb]-PSMA in LNCaP and PC3 cells was approximately 40% for 4 hours. However, since PC3 is an androgen receptor-negative cell line, PSMA uptake was not expected. For this reason, further studies are planned to confirm these results. According to 2-way ANOVA for the optimum time of cell retention, a significant difference was found between [^161^Tb]TbCl_3_ and [^161^Tb]-PSMA-617 in LNCaP and PC3 cells. *In vitro* studies comparing [^161^Tb]-PSMA-617 and [^177^Lu]-PSMA-617 in the literature have noted that [^161^Tb]-PSMA-617 showed 3 times more uptake compared to [^177^Lu]Lu-PSMA-617 in the PC3-PIP cell line (Gracheva et al., 2019), probably due to the incorporation of the PSMA-617 peptide by the cells and the Auger electrons emitted by [^161^Tb]Tb (Müller et al., 2014; Müller, Umbricht, et al., 2019).

Scintigraphy imaging visualized the accumulation of [^161^Tb]-PSMA-617 in the prostate, kidneys, and the bladder. Static images of [^161^Tb]-PSMA-617 in rats the substantial tracer accumulation was present in the kidneys at 30 min. In addition, [^161^Tb]-PSMA-617 activity in the abdominal and chest region also increased with time. [^161^Tb]-PSMA-617 activity was almost entirely excreted after 4 h by renal excretion. Our results here are similar to Müller et al.’s study (Müller, Umbricht, et al., 2019), where SPECT/CT images were obtained of PC-3 PIP/flu tumor-bearing mice at 1 h, 4 h, and 24 h after being injected with ~ 25 MBq [^161^Tb]-PSMA-617. In that study, while [^161^Tb]-PSMA-617 accumulated in the PIP-3 tumor xenograft on the right side, there was only negligible uptake in the PSMA-negative PC-3 flu tumor on the left side. Like LNCaP cells in our study, PC3-PIP cells are androgen receptor cells for which PSMA-617 exhibits higher affinity, explaining the accumulation of [^161^Tb]-PSMA-617 on the right side. They also reported that renal excretion of [^161^Tb]-PSMA-617 was rapid, with almost the entire activity excreted within 4 hours.

Biodistribution results (Fig. 7) were compatible with the imaging results. Biodistribution of [^161^Tb]-PSMA-617 in Albino Wistar rats revealed a notable concentration of the compound within a 24-hour timeframe in the renal, vesicular, and urinary compartments. This specific inclination toward renal tissues underscores the dominant route of excretion for [^161^Tb]-PSMA-617 being through the kidneys. Hematological dynamics displayed an initial surge over a 24-hour period, followed by a subsequent reduction at the 48-hour mark. The identifiable cause for this trend lies in the noticeable absence of an established tumor model within the experimental group of Albino Wistar rats. In contrast, Müller et al.’s study (Müller, Umbricht, et al., 2019) which involved well-established tumor models consistently demonstrated a declining trajectory in systemic [^161^Tb]-PSMA-617 levels, as evidenced by the blood-tumor ratio. In our study, at the 24-hour time point, a marked increase in fecal content was observed, while at the subsequent 48-hour time point, a statistically significant elevation was noted in specific anatomical sites, including the pancreas, musculature, adipose tissue, salivary glands, and thyroid. The presence of PSMA accumulation in salivary glands is a known phenomenon in PSMA-related research, justifying the routine clinical application of cold compress therapy during the course of treatment. Similarly, the upsurge in fecal levels is interpreted as an indicative outcome of PSMA excretion via the fecal route. Furthermore, a gradual increase in prostatic tissue uptake was distinctly observed over the initial 24-hour window. In contrast, minimal alterations were observed across other tissue types.

## Conclusions

While there have only been a few studies on [^161^Tb]-PSMA-617 for the treatment of prostate cancer in the literature, the remarkable results obtained thus far in this study and in the literature may encourage more interest in [^161^Tb]-PSMA-617. Specifically, this preclinical study will pave the way for further preclinical research activities on [^161^Tb]-PSMA-617 by our research team, with the aim of clinical applications in the near future to benefit patients with metastatic metastatic castration resistant prostate cancer.

## Abbreviations

Cd(Te): Cadmium telluride
DOTA 1,4,7,10: Tetraazacyclododecane-1,4,7,10-tetraacetic acid
DOTAGA 2,2′,2”: (10-(2,6-dioxotetrahydro-2H-pyran-3-yl)-1,4,7,10-tetraazacyclododecane-1,4,7-triyl)triacetic acid
HPLC: High Performance Liquid chromatography
PARP: poly ADP ribose polymerase
PC: Prostate Cancer
PSMA: Prostate specific membran antigen
ROI: Region of interest
SPECT: Single Photon Emission Computed Tomography
TLC: Thin liquid chromatography
